# Comparable long term psychosocial burden in patients with lower grade versus higher grade brain tumors

**DOI:** 10.1038/s41598-025-11456-2

**Published:** 2025-07-18

**Authors:** Simone D’Souza, Stefanie Fuchs, Marco Skardelly, Stephan Zipfel, Björn Falkenburger, Martin Teufel

**Affiliations:** 1 Department of Neurology, Goethe University Frankfurt, University Hospital, Frankfurt am Main, Germany; 2 Department of Psychosomatic Medicine and Psychotherapy, University Medical Hospital Tübingen, Tübingen, Germany; 3 Department of Neurology, Faculty of Medicine, University Hospital Carl Gustav Carus, TU Dresden University of Technology, Dresden, Germany; 4 Center for Neuro-Oncology, Comprehensive Cancer Center Tübingen- Stuttgart, University Hospital Tübingen, Tübingen, Germany; 5 Department of Neurosurgery, University Hospital Tübingen, Tübingen, Germany; 6 Section of Psycho-Oncology, West German Cancer Center (WTZ), University Hospital Duisburg-Essen, Essen, Germany; 7 Department of Psychosomatic Medicine and Psychotherapy, LVR University Hospital Duisburg-Essen, Essen, Germany; 8 German Centre of Mental Health (DZPG), University of Tübingen, Tübingen, Germany

**Keywords:** Brain tumor, Hope, Depression, Anxiety, Distress, Psychosocial counseling, CNS cancer, Comorbidities

## Abstract

Patients with higher grade brain tumors (PwHG) frequently experience anxiety and depression, while patients with lower grade brain tumors (PwLG) may also develop these issues over time. However, it remains unclear whether PwLG face additional psychosocial challenges, such as reduced hope, heightened distress, or impaired coping, compared to PwHG. This study aimed to comparatively analyze hope, distress, coping, anxiety, and depression in PwLG versus PwHG to inform psychosocial screening and care. A total of 66 patients were assessed using the Distress Thermometer (DT), Generalized Anxiety Disorder questionnaire-2 (GAD-2), Patient Health Questionnaire-2 (PHQ-2), Herth Hope Index (HHI), and a self-developed coping questionnaire across three time points. Repeated-measures ANOVAs, post-hoc Bonferroni tests, and Friedman tests were conducted. Results revealed no significant group differences in distress, anxiety, or depression. PwLG exhibited a decline in hope (*p* = 0.044) and treatment success expectations (*p* = 0.018) over time, mirroring patterns observed in PwHG. These findings indicate that PwLG and PwHG face comparable psychosocial burdens, suggesting that similar psychosocial interventions may benefit both groups.

**Supplementary Information:**

The online version contains supplementary material available at 10.1038/s41598-025-11456-2.

## Introduction

Glioblastoma is the most lethal form of cancer among neuro-oncological diseases^1^. Annually, approximately five per 100,000 residents are newly affected^2^while the median survival time is approximately 20 months, despite the administration of maximal therapy^3,4^. Patients are typically in the prime of their lives when they receive a diagnosis of this disease^4,5^. The psychological burden on these patients is significant^6^. Therefore, medical treatment, as a standard, should also include screening for mental comorbidities as a routine component^7^. Patients with another higher grade brain tumor, such as an astrocytoma, may have a higher survival rate^8^. However, the psychological burden of the disease and therapy-related distress remain significant concerns^9^.

Conversely, patients with a meningioma or an alternative lower grade brain tumor frequently demonstrate a greater longevity than patients with a higher grade brain tumor^10^. However, the psychological well-being of these patients was also described as impaired^11–13^. Currently, there is no insight into the difference in longitudinal mental burden in terms of hope, distress, and coping parameters in addition to depression and anxiety in patients with a higher grade brain tumor compared to patients with a lower grade brain tumor.

Based on the more aggressive course and limited prognosis associated with higher grade tumors^14^ we hypothesized that patients with higher grade brain tumors would report a greater and more persistent psychological burden—particularly lower levels of hope—over the course of the disease compared to patients with lower grade tumors.

Consequently, the objective of this study was to conduct a comparative analysis of three consecutive measurement points of patients with lower grade (PwLG) and higher grade brain tumors (PwHG) with regard to psychosocial distress, anxiety, depression, hope, and coping parameters.

## Methods

### Patients

This longitudinal study was conducted with three successive measurement points in patients with a brain tumor (PwBT) from the Neurosurgical Clinic of the University Hospital of Tübingen. The recruitment took place between September 2015 and November 2016. A preliminary account of the data from the initial measurement point has already been published^15^. Inclusion criteria were: (1) sufficient cognitive ability to complete the questionnaires according to the treating physician, (2) proficiency of written and spoken German. (3) no schizophrenia or florid delusional disorder unrelated to the neurological disease (per patient file). Exclusion criteria were: (1) brain metastases originating from primary tumors in other organs (e.g., malignant melanoma, breast cancer, or lung cancer); (2) unclear tumor histology; and (3) presence of multiple brain tumors. The patients were introduced to the study in the outpatient clinic waiting room. After giving informed consent, the participants were provided a questionnaire package via tablet PC that included the Patient Health Questionnaire-2 (PHQ-2), Generalized Anxiety Disorder-2 (GAD-2), Herth Hope Index (HHI), Distress Thermometer (DT), a self-developed paper-pencil questionnaire for the assessment of coping parameters, and questions assessing demographic and clinical data.

Seventy-one patients were consecutively assessed, with five patients excluded (one brain metastasis in renal cell carcinoma, two adenoma of the adenohypophysis, one unclear mass without biopsy, one schizophrenia). Sixty-six patients were assessed and included in the analysis. The patients gave their consent and completed the questionnaire on a tablet PC in the hospital.

The study employed a longitudinal design with three measurement time points (T0-T2). The first measurement time point (T0) was conducted perioperatively to establish baseline data, with 61 of the 66 enrolled patients completing the survey one day prior to surgery, and 5 patients being assessed four to six days postoperatively due to logistical constraints. The second measurement time point (T1) was scheduled to coincide with the early postoperative evaluation, the planning of potential adjuvant chemotherapy, and the first follow-up MRI^14^. At this time point, 46 patients completed the assessment, approximately three months after surgery (mean = 110.15 days, SD = 106.71). The third measurement time point (T2) focused on long term adjustment phase and was conducted approximately one year after surgery, with 19 patients participating (mean = 327.47 days after the initial assessment, SD = 127.02). As assessments were aligned with patients’ scheduled hospital visits, some variability in timing occurred due to logistical and clinical factors.

The study was conducted in accordance with relevant guidelines and regulations. Ethics approval was obtained beforehand from the local ethics committee of the Medical Faculty at the University of Tübingen (reference number 602/2014BO2).

### Assessment instruments

PwBT were administered the Distress Thermometer (DT), the Generalized Anxiety Disorder-2 (GAD-2), the Patient Health Questionnaire-2 (PHQ-2), the Herth Hope Index (HHI), a questionnaire on demographic and clinical data via tablet PC, and a self-developed questionnaire on coping parameters as a paper-pencil questionnaire. The German versions of all questionnaires were used.

The Distress Thermometer (DT) is a scale with values ranging from 0 to 10. A score of zero indicates no distress, while a score of 10 indicates maximum distress^16–18^. Patients are instructed to indicate which value best describes their level of distress during the previous week. Furthermore, the DT comprises a list of 34 items pertaining to various domains, including practical, emotional, familial, spiritual, and physical problems^19,20^. A score of ≥ 5 is indicative of a patient exhibiting symptoms above the threshold level^21^. The DT is a valid and reliable screening instrument, exhibiting 83% sensitiviy and 68% specificiy^22^.

The Generalized Anxiety Disorder-2 (GAD-2) is a brief screening tool comprising two items designed to assess the presence of generalized anxiety disorder^23^. The total score on the GAD-2 ranges from 0 to 6, with higher scores indicating greater severity of anxiety. Higher scores are indicative of elevated anxiety levels. A value of 3 or higher is considered a significant indicator of pathological anxiety^23,24^. The GAD-2 has been demonstrated to perform well as a screening tool for anxiety disorders, with an area under the curve ranging from 0.80 to 0.91^24^. It is particularly well-suited for use in patients with brain tumors. As evidenced by studies on neurological patients (Cronbach’s α = 0.77)^25^ and head and neck cancer patients (Cronbach’s α = 0.87)^26^the GAD-2 demonstrates acceptable and good internal consistency, respectively.

The Patient Health Questionnaire-2 (PHQ-2) is a brief screening tool consisting of two items designed to assess the presence of depressive symptoms^27^. The combined score of the two questions (generated by addition) ranges from 0 to 6, with higher scores indicating a greater severity of depressive symptoms. A cut-off score of ≥ 3 is optimal for screening for depression. The PHQ-2 has a sensitivity of 87%and a specificity of 78%for the diagnosis of major depressive disorder^27^. Preliminary results indicate that the PHQ-2 is an effective tool for identifying depression in individuals diagnosed with brain tumors, with a moderate level of internal consistency (Cronbach’s alpha = 0.68)^28^.

The Herth Hope Index (HHI) is a validated and reliable tool for measuring hope in both research and clinical settings. It consists of 12 items on a 4-point Likert scale, with scores ranging from 12 to 48, with higher scores indicating greater hope^29^. The HHI has high practicability, robust internal consistency (Cronbach’s α = 0.82), and substantial concurrent validity, particularly in the context of cancer patients^30^.

The self-developed Individual Coping Questionnaire, previous published elsewhere^15,31^consists of the following six 10-point Likert-scale aspects: (1) expectations of treatment success (1 = full recovery; 10 = no recovery), (2) expectations of side effects (1 = good; 10 = bad), (3) former resilience rating (1 = very strong; 10 = non-existent), (4) current subjective state of mental health (1 = not limited; 10 = limited), (5) ability to handle the disease (1 = good; 10 = bad), and (6) social environment support (1 = extensively available; 10 = significantly reduced).

### Clinical data

The grouping of the tumor entities at the initial measurement point have been previously described in detail elsewhere^15^.

### Statistical analyses

Statistical analysis was performed using JASP version 0.18.3 (Apple Silicon). Power analysis has been carried out by G*Power Version 3.1. Socio-demographic, clinical and psychosocial data were presented descriptively. To assess the development of distress, anxiety, depression, hope and coping parameters over time, repeated measures ANOVAS were calculated for the entire study sample. Greenhouse-Geisser adjustment was used to correct sphericity violations. The post-hoc tests were carried out with the Bonferroni correction. Due to a low number of valid cases at the third measurement point (*N* = 14 patients with a lower grade tumor and *N* = 5 patients with a higher grade tumor), non-parametric Friedman tests were calculated. Effect sizes were calculated using partial eta-squared (η²) for repeated measures ANOVAs. Given the exploratory nature of the study and the reduced sample size at follow-up, effect sizes were reported alongside p-values to support a more nuanced interpretation of the results and to account for the limited statistical interpretation^32,33^. According to Cohen, values of η² = 0.01, 0.06, and 0.14 are interpreted as small, medium, and large effects, respectively^32^. To evaluate potential systematic differences between completers and non-completers, baseline sociodemographic, clinical, and psychosocial characteristics were compared using chi-square tests. For a preliminary reliability analysis of the self-developed coping questionnaire, Cronbach’s alpha was calculated. Values above 0.80 are generally considered indicative of good internal consistency^34^. A p - value of less than 0.05 was considered statistically significant.

## Results

The sociodemographic, clinical, and psycho-social data collected at the initial measurement point have been previously described in detail in a separate publication^15^ (Table 1 and Supplementary Table 1).

**Table 1:** Table legend: demographic characteristics of patients with lower grade and higher grade brain tumors at three measurement points (T1, T2, T3).

Lower grade/higher grade brain tumor			
	T 1 (*N* = 43/23)	T 2 (*N* = 33/13)	T 3 (*N* = 14/5)
**Age (SD)**	53.7 (11.1)/51.2 (14.7)	54.2 (10.6)/48.2 (13.8)	53.2 (18.8)/46 (11.7)
	**Number (Percent)**	**Number (Percent)**	**Number (Percent)**
**Sex**			
Female	30 (69.8)/8 (34.8)	20 (60.6)/3 (23.1)	7 (50)/2 (60)
Male	13(30.2)/15 (65.2)	13 (39.4)/10 (76.9)	7 (50)/3 (40)
**Family status**			
Living in partnership	32 (74.4)/19 (82.6)	26 (78.8)/10 (76.9)	11 (78.6)/4 (80)
Living alone	3 (7.0)/3 (13.0)	4 (12.1)/3 (23.1)	2 (14.3)/1 (20)
Other	8 (18.6)/1 (4.3)	3 (9.1)/0 (0)	1 (7.1)/0 (0)
**Living situation**			
Alone	8 (18.6)/1 (4.3)	6 (18.2)/0 (0)	3 (21.4)/0 (0)
With partner, child(ren), parents	31 (72.1)/21 (91.3)	27 (81.8)/13 (100)	11 (78.6)/5 (100)
Alone with child(ren)	2 (4.7)/0 (0)	0 (0)/0 (0)	0 (0)/0 (0)
Other	2 (4.7)/1 (4.3)	0 (0)/(0)	0 (0)/0 (0)
**Children**			
Yes	36 (83.7)/14 (60.9)	30 (90.9)/7 (53.8)	11 (78.6)/3 (60)
No	7 (16.3)/9 (39.1)	3 (9.1)/6 (46.2)	3 (21.4)/2 (40)
**Education**			
< 10 year	9 (20.9)/12 (52.2)	7 (21.2)/6 (46.1)	3 (21.4)/0 (0)
≥ 10 years	21 (48.9)/18 (78.3)	18 (54.6)/3 (23.1)	6 (42.9)/2 (20)
University degree	11 (25.6)/3 (13)	7 (21.2)/3 (23.1)	4 (28.6)/2 (40)
Other	2 (4.7)/1 (4.3)	1 (3.0)/1 (7.7)	1 (7.1)/1 (20)

### DT

The DT value exhibited moderately elevated levels in both groups throughout the course of the disease (Supplementary Fig. 1). Repeated-measures ANOVA revealed no statistically significant difference in the disease trajectory in mean DT levels (F(2,34) = 0.833, *p* = 0.444, partial η² = 0.23), corroborated by the non-parametric Friedman Test (*p* = 0.209). No statistically significant difference was observed in mean DT levels between the two groups of patients with higher grade and lower grade brain tumors during the disease trajectory (F(1,17) = 0.473, *p* = 0.501, partial η² = 0.014). Bonferroni-adjusted post-hoc analysis revealed no statistically significant difference in DT levels between the two groups (*p* = 0.501, *M*_Diff_ = − 0.705, 95% CI [− 2.876, 2.464]), nor in the disease trajectory (e.g. T0 vs. T2: *p* = 1.00, *M*_Diff_ = 0.314, 95% CI [− 1.835, 4.013]).

### GAD – 2

The GAD − 2 level remained stable throughout the disease trajectory in both groups (Supplementary Fig. 1). Repeated-measures ANOVA revealed no statistically significant difference in the disease trajectory in mean GAD − 2 levels (F(2,34) = 1.220, *p* = 0.308, partial η² = 0.0029), corroborated by the non-parametric Friedman Test (*p* = 0.341). No statistically significant difference was observed in mean GAD − 2 levels between the two groups of patients with higher grade and lower grade brain tumors (F(1,17) = 0.141, *p* = 0.712, partial η² = 0.004). Bonferroni-adjusted post-hoc analysis revealed no statistically significant difference in GAD-2 levels between the two groups (*p* = 0.712, *M*_Diff_ = − 0.257, 95% CI [− 1.702, 1.187]), nor in the disease trajectory (e.g. T0 vs. T2: *p* = 1.00, *M*_Diff_ = − 0.021, 95% CI [− 1.345, 1.302]).

### PHQ – 2

No appreciable decline in the PHQ − 2 score was observed in both groups throughout the disease trajectory (Supplementary Fig. 1). Repeated-measures ANOVA revealed no statistically significant difference in the disease trajectory in mean PHQ − 2 levels (F(2,34) = 0.398, *p* = 0.675, partial η² = 0.009), corroborated by the non-parametric Friedman Test (*p* = 0.167). No statistically significant difference was observed in mean PHQ − 2 levels between the two groups of patients with higher grade and lower grade brain tumors (F(1,17) = 2.147, *p* = 0.706, partial η² = 0.005). Bonferroni-adjusted post-hoc analysis revealed no statistically significant difference in PHQ − 2 levels between the two groups (*p* = 0.706, *M*_Diff_ = 0.248, 95% CI [− 1.114, 1.610]), nor in the disease trajectory (e.g. T0 vs. T2: *p* = 1.00, *M*_Diff_ = 0.071, 95% CI [− 1.110, 1.252]).

### HHI

A noteworthy decline in the level of hope was observed in both groups throughout the disease trajectory (Supplementary Fig. 1). Repeated-measures ANOVA revealed a statistically significant difference in the disease trajectory in mean HHI levels, F(2,10) = 4.341, *p* = 0.044, partial η² = 0.20, corroborated by the non-parametric Friedman Test (*p* = 0.044). No statistically significant difference was observed in mean HHI levels between the two groups of patients with higher grade and lower grade brain tumors during the disease trajectory (F(1,5) = 2.476, *p* = 0.176, partial η² = 0.158). Bonferroni-adjusted post-hoc analysis revealed a statistically significant reduction (*p* = 0.044) in HHI levels in the entire study sample between measurement points 1 and 2 (*M*_Diff_ = 5.54, 95% CI [0.13, 10.95]), while no significant difference was detected between the two groups (*p* = 0.176, *M*_Diff_ = − 4.028, 95% CI [− 10.608, 2.552])

### Individual coping questionnaire items

#### Expectations treatment success

As the disease progressed, the item “expectations treatment success” declined to a marked degree in both groups (Supplementary Fig. 2). Repeated-measures ANOVA revealed a statistically significant difference in “expectations treatment success” levels across disease progression, F(2,12) = 5.102, *p* = 0.025, partial η² = 0.273, corroborated by the non-parametric Friedman Test (*p* = 0.018). Furthermore, no statistically significant difference was observed in the mean “expectations treatment success” levels between the two groups of patients with higher grade and lower grade brain tumors (F(1,6) = 0.170, *p* = 0.695, partial η² = 0.010). A Bonferroni-adjusted post-hoc analysis revealed a statistically significant difference in “expectations treatment success” levels between measurement point 1 and measurement point 3 (*p* = 0.027, *M*_Diff_ = −3.267, 95% CI [−6.187, −0.346]), no significant difference was detected between the two groups (*p* = 0.695, *M*_Diff_ = 0.511, 95% CI [− 2.526, 3.548]).

#### Expectations side effects

There was no notable increase in the item “expectations side effects” observed in both groups throughout the disease trajectory (Supplementary Fig. 2). Repeated-measures ANOVA revealed no statistically significant difference in the disease trajectory in mean “expectations side effects” levels (F(2,12) = 0.056, *p* = 0.378, partial η² = 0.053), corroborated by the non-parametric Friedman Test (*p* = 0.902). No statistically significant difference was observed in mean “expectations side effects” levels between the two groups of patients with higher grade and lower grade brain tumors (F(1,6) = 0.115, *p* = 0.746, partial η² = 0.010). Bonferroni-adjusted post-hoc analysis revealed no statistically significant difference in “expectations side effects” levels between the two groups (*p* = 0.746, *M*_Diff_ = 0.322, 95% CI [− 2.003, 2.648]), nor in the disease trajectory (e.g. T0 vs. T2: *p* = 1.00, *M*_Diff_ = 0.384, 95% CI [− 1.344, 2.111]).

#### Former resilience rating

The assessment of former resilience was found to be favorable in both groups throughout the course of the disease (Supplementary Fig. 2). Repeated-measures ANOVA revealed no statistically significant difference in the disease trajectory in mean “former resilience rating” levels (F(2,8) = 1.417, *p* = 0.300, partial η² = 0.123), corroborated by the non-parametric Friedman Test (*p* = 0.56). No statistically significant difference was observed in mean “former resilience rating” levels between the two groups of patients with higher grade and lower grade brain tumors (F(1,4) = 0.914, *p* = 0.393, partial η² = 0.098). Bonferroni-adjusted post-hoc analysis revealed no statistically significant difference in the item “former resilience rating” between the two groups (*p* = 0.393, *M*_Diff_ = − 0.917, 95% CI [− 3.579, 1.745]), nor in the disease trajectory (e.g. T0 vs. T2: *p* = 0.399, *M*_Diff_ = − 1.250, 95% CI [− 3.504, 1.004).

#### Subjective state mental health

The item “subjective state mental health” of both groups was evaluated to be moderate throughout the course of the disease (Supplementary Fig. 2). Repeated-measures ANOVA indicated that there was no statistically significant difference in the disease trajectory in the item “subjective state mental health” (F(2,12) = 0.241, *p* = 0.789, partial η² = 0.016), corroborated by the non-parametric Friedman Test (*p* = 0.639). No statistically significant difference was observed in “subjective state mental health” between the two groups of patients with higher grade and lower grade brain tumors (F(1,6) = 0.862, *p* = 0.389, partial η² = 0.069). Bonferroni-adjusted post-hoc analysis revealed no statistically significant difference in the item “subjective state mental health” between the two groups (*p* = 0.389, *M*_Diff_ = 1.000, 95% CI [− 1.636, 3.636]), nor in the disease trajectory (e.g. T0 vs. T2: *p* = 1.000, *M*_Diff_ = − 0.567, 95% CI [− 2.938, 1.805).

#### Ability to handle the disease

The item “ability to handle the disease” of both groups was found to be consistently good throughout the course of the disease, with an observed increase at the third measurement point towards a less ability in patients with higher grade brain tumors that did not reach statistical significance (Supplementary Fig. 2). Repeated-measures ANOVA indicated that there was no statistically significant difference in the disease trajectory in the item “ability to handle the disease”, F(2,12) = 3.226, *p* = 0.076, partial η² = 0.136, corroborated by the non-parametric Friedman Test (*p* = 0.191). No statistically significant difference was observed in the item “ability to handle the disease” between the two groups of patients with higher grade and lower grade brain tumors (F(1,6) = 3.093, *p* = 0.129, partial η² = 0.161). Bonferroni-adjusted post-hoc analysis revealed no statistically significant difference in the item “ability to handle the disease” between the two groups (*p* = 0.129, *M*_Diff_ = 1.600, 95% CI [− 0.626, 3.826]), nor in the disease trajectory (e.g. T0 vs. T2: *p* = 0.089, *M*_Diff_ = − 1.750, 95% CI [− 3.723, 0.223).

#### Social environment support

The item “social environment support” was rated as positive for both groups throughout the course of the disease (Supplementary Fig. 2). Repeated-measures ANOVA indicated that there was no statistically significant difference in the disease trajectory in the item “social environment support” (F(2,12) = 2.646, *p* = 0.112, partial η² = 0.158), corroborated by the non-parametric Friedman Test (*p* = 0.156). No statistically significant difference was observed in the item “social environment support” between the two groups of patients with higher grade and lower grade brain tumors (F(1,6) = 0.448, *p* = 0.528, partial η² = 0.158). Bonferroni-adjusted post-hoc analysis revealed no statistically significant difference in experienced social support rating between the two groups (*p* = 0.528, *M*_Diff_ = 0.233, 95% CI [− 0.620, 1.086]), nor in the disease trajectory (e.g. T0 vs. T2: *p* = 0.929, *M*_Diff_ = − 0.300, 95% CI [− 1.086, 0.486).

Chi-squared tests comparing baseline characteristics of completers and non-completers revealed no significant differences in sociodemographic (female vs. male: χ²(1) = 0.30, *p* = 0.584, φ = −0.08; family status: χ²(1) = 0.81, *p* = 0.667, φ = 0.13; living situation χ²(1) = 2.29, *p* = 0.51, φ = 0.22; children χ²(1) = 0.94, *p* = 0.333, φ = −0.14; and education χ²(1) = 1.58, *p* = 0.664, φ = 0.19), clinical (HG vs. LG χ²(1) = 0.06, *p* = 0.806, φ = 0.04), or psychosocial measures (DT positive vs. DT negative: χ²(1) = 0.26, *p* = 0.607, φ = −0.076; GAD-2 positive vs. GAD-2 negative: χ²(1) = 0.40, *p* = 0.528, φ = −0.09; PHQ-2 positive vs. PHQ-2 negative: χ²(1) = 0.05, *p* = 0.831, φ = −0.03; median-split between HHI scores ≥ 44 vs. HHI scores ≤ 43: χ²(1) = 0.003, *p* = 0.958, φ = 0.01). We found no specific dropout patterns, supporting the assumption of data missing at random.

In addition, a subsequent chi-squared test revealed a significantly higher proportion of female compared to male patients in the PwLG group (χ²(1) = 7.51, *p* = 0.006, φ = 0.34).

In order to conduct preliminary reliability analysis of the individual coping questionnaire, Cronbach’s alpha was computed to assess the internal consistency of the subscales, which consists of six items. The internal consistency was found to be good, with a Cronbach’s alpha of 0.811.

Given the exploratory nature of the study and the observed drop-off over time, a post-hoc power analysis was conducted to estimate the sensitivity of the repeated measures ANOVA in detecting time effects. Assuming a medium effect size (f = 0.25), α = 0.05 and the actual sample sizes over time, the power to detect a main effect of time was approximately 0.88. However, due to the small sample sizes at later time points (*N* = 14 PwLG, *N* = 5 PwHG) the power for group comparisons – especially at the third measurement point – was significantly reduced, which limited the ability to detect smaller effects with sufficient certainty.

## Discussion

The objective of the study was to compare the experience of distress, anxiety, depression, and coping parameters between PwLG and PwHG over the course of the disease, assessed at three successive time points: baseline (perioperative), short-term follow-up (approximately three months postoperatively), and long term follow-up (approximately one year after surgery). Based on the poorer prognosis and more aggressive disease trajectory, we hypothesized that PwHG would show persistently lower levels of hope and higher psychological burden over time compared to PwLG. However, the data indicate that PwLG similar to PwHG experience a significant decline in hope, comparable psycho-oncological distress and emotional challenges like anxiety, depression, accompanied by a significant decline in expectations regarding treatment success approximately one year after surgery.

Interestingly, both PwHG and PwLG reported comparable levels of psychological burden over the course of the disease, including a decline in hope and treatment expectations. This result is somewhat counterintuitive, as one might expect patients with a less severe diagnosis to maintain higher levels of hope and more favorable treatment expectations approximately one year after surgery, particularly in light of evidence showing that patients with tumor recurrence report lower hope and greater mood disturbance compared to those who are newly diagnosed or recurrence-free^35^. However, previous studies have shown that PwLG also face increased rates of anxiety and depression^12,13^. While it is known that patients with glioblastoma are highly likely to experience significant psycho-oncological distress^36^, the mechanisms underlying the absence of significant differences in psychological burden between the two groups remain unclear. Several hypotheses may help explain the underlying mechanisms.

First, the subjective perception of the disease by the patients themselves might play a crucial role. Both groups underwent neurosurgical procedures (either craniotomy or biopsy), which is associated with anxiety and a sense of loss of control^13^. It may be assumed that the specific type of brain tumor may not initially influence the subjective perception of patients, as they are typically not medical professionals. The present findings may support this assumption, as PwLG also exhibited a decline in expectations regarding treatment success over time, similar to PwHG. One possible explanation could be that the mere knowledge of an intracranial growth—regardless of tumor grade—could evoke a general sense of loss of control, potentially contributing to a decline in hope and an increase in psycho-oncological distress, anxiety, and depression. However, this hypothesis remains speculative and should be examined in future studies.

Second, meningioma is more prevalent in women – including in the present sample – which is likely attributable to hormonal factors^37^. Women are more likely to experience psychological burden, including anxiety and depression^38,39^. A possible explanation for the increased psychosocial distress observed in PwLG could therefore be the overrepresentation of female patients in this group.

Third, based on previous literature^40,41^PwHG may underestimate their mental burden due to limited symptom awareness and impaired emotion recognition.

Bunevicius and colleagues have pointed out that the benefits of continuous screening of patients with a meningioma are still uncertain^13^. Nevertheless, we believe it is reasonable to assume that the benefits of tablet-based screening on admission or during an outpatient appointment outweigh the effort. We consider it is imperative to determine the psycho-oncological burden of both PwLG and PwHG in order to ensure an appropriate treatment plan. Recently published studies indicate a significantly increased risk of suicide attempts in patients with neurological impairment^42^ especially in male patients with a glioblastoma^43^. In addition, depression and suicidal risk screening in adults is recommended, despite the lack of evidence of a fair balance of benefits and harms of screening^44^.

Moreover, the decline in hope observed in this study one year after diagnosis in both PwHG and PwLG might indicate a need for more comprehensive palliative care and end-of-life discussions in PwHG^45,46^as well as an enhanced psychosocial support for PwLG. However, these interpretations are preliminary should be explored further in future studies.

This study has limitations. (1) Due to the relatively modest number of participants, particularly at the final measurement point, it is not possible to draw any definitive conclusions. To address this limitation, non-parametric tests were applied as suitable statistical approach. Nonetheless, the confidence intervals from selected post-hoc analyses indicate meaningful trends over time. (2) The questionnaire on coping is a self-developed instrument that has yet to undergo the requisite validation process. Consequently, the results must be interpreted with caution and only limited conclusions can be drawn. However, preliminary reliability testing indicated good internal consistency (Cronbach’s alpha = 0.811). (3) A noteworthy increase was observed in the parameter “ability to handle the disease” (range from “good” to “bad”) of the questionnaire pertaining to coping parameters in PwHG. However, this did not achieve statistical significance, which would be anticipated with a larger sample size. (4) The location of the tumor may influence the response behavior^47,48^. It is possible that the decline in hope in PwHG is related to tumor location, as the highest proportion of PwHG had a left-sided brain tumor at the last measurement point. However, due to the small sample size, this hypothesized correlation should be investigated further in a larger study. (5) In line with the exploratory nature of the study, we retained a significance level of 0.05 and did not apply a global correction for multiple testing. As such, the findings should be considered with caution rather than as confirmatory evidence. (6) A complete-case analysis was applied, assuming data were missing at random based on baseline comparisons. While this assumption was supported by statistical comparisons, selective dropout cannot be entirely ruled out.

In conclusion, this study provides insights into the psychological burden of patients with both lower grade and higher grade brain tumors over the course of their disease. By employing a longitudinal design with three measurement points over one year, the study enables the tracking of changes in levels of distress, anxiety, depression, hope, and coping over time. The observed decline in hope and treatment expectations, along with persistently high levels of distress in both groups, underscores the importance of continuous psychosocial support regardless of tumor grade. These findings highlight the importance of not underestimating the psychological burden in patients with lower grade tumors, and support the implementation of standardized psycho-oncological assessments across all tumor grades.

## Electronic supplementary material

Below is the link to the electronic supplementary material.

Supplementary Material 1

## Data Availability

Data and analytical methods used in this study are available from the corresponding author, Simone D’Souza (dr.simone.dsouza@gmail.com), upon reasonable request.
