# Supplementary material for: Comparable long term psychosocial burden in patients with lower grade versus higher grade brain tumors
Source: Sci Rep. 2025 Jul 18;15:26089. doi: 10.1038/s41598-025-11456-2 (PMC12274605; doi:10.1038/s41598-025-11456-2)
Supplement: Supplementary file 1 — Supplementary Material 1 [file 41598_2025_11456_MOESM1_ESM.docx]

Supplementary Figure 1.

Supplementary Figure legend 1: Distress Thermometer (DT), Generalized Anxiety Disorder-2 (GAD-2), Patient Health Questionnaire-2 (PHQ-2), and Herth Hope Index (HHI) scores over time. Error bars represent standard errors.


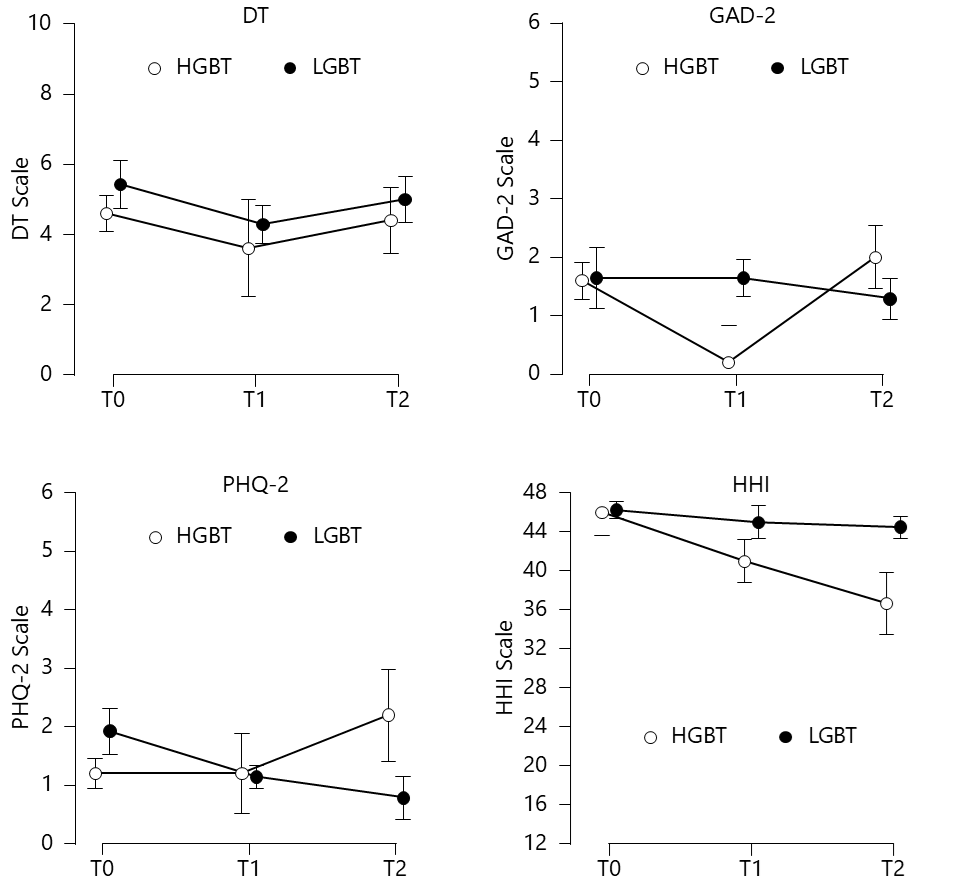


Supplementary Figure 2.

Supplementary Figure legend: Individual Coping Questionnaire scores over time for six coping aspects: treatment success, side effects, resilience, mental health, disease handling, and social support. Error bars represent standard errors.
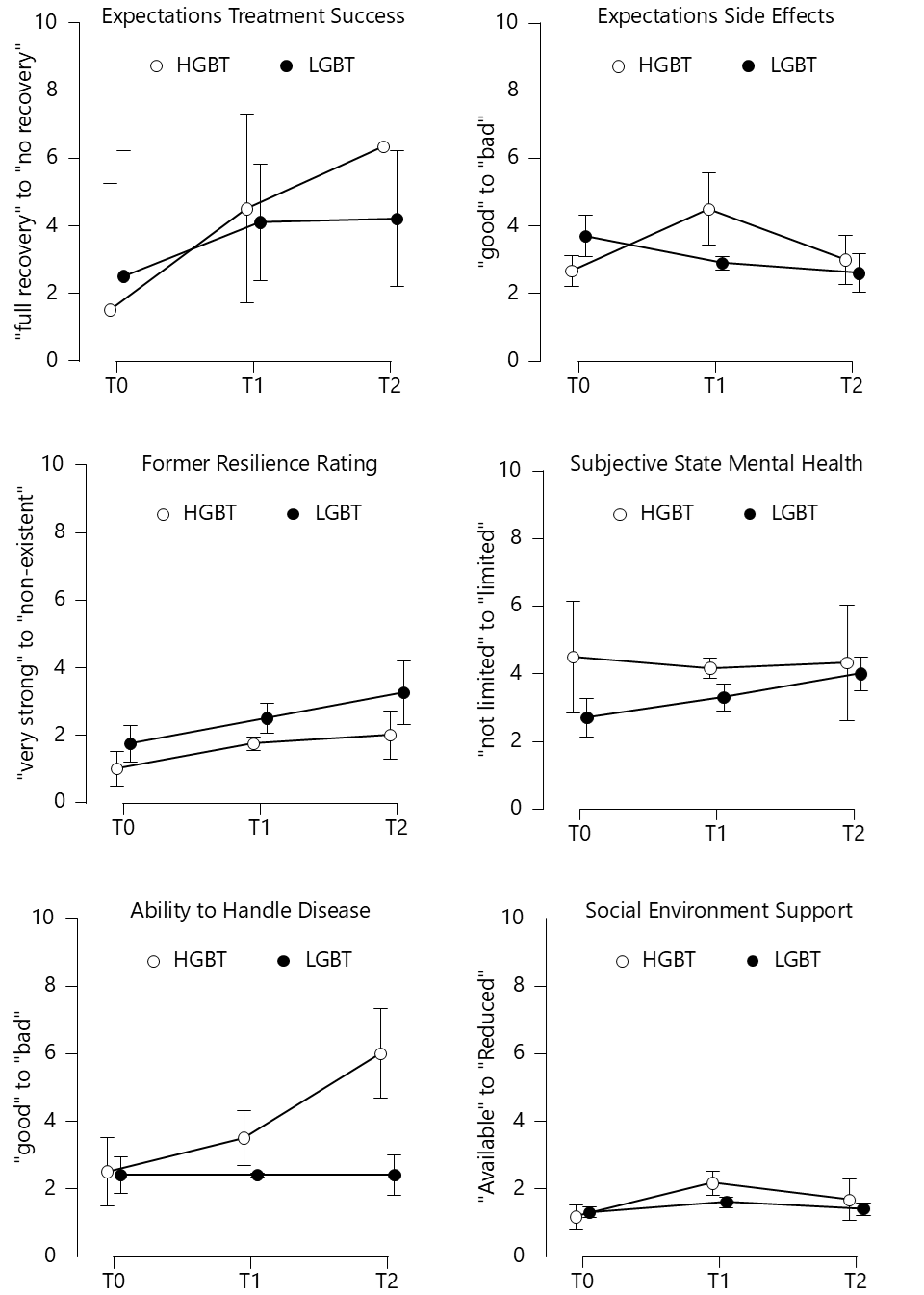


Supplementary Table 1. Clinical and Psychosocial Data

Supplementary Table 1. Legend: Clinical and Psychosocial Data collected at three measurement points, including distress, anxiety, depression, hope, and coping parameters.

| **Supplementary Table 1. Clinical and Psychosocial Data** | | | |  |
| --- | --- | --- | --- | --- |
|  | **Lower-grade / higher-grade brain tumor** | | |  |
|  | **T 1 (N = 43/23)** | **T 2 (N = 33/13)** | **T 3 (N = 14/5)** |  |
|  | **Number (Percent)** | **Number (Percent)** | **Number (Percent)** | **p-value** |
| **Localization** |  |  |  |  |
| Right | 21 (48.8) / 12 (52.2) | 15 (45.5) / 5 (38.5) | 4 (28.6) / 0 (0) |  |
| Left | 18 (41.9) / 9 (39.1) | 15 (45.5) / 6 (46.2) | 7 (50.0) / 3 (60) |  |
| Not localizable | 4 (9.3) / 2 (8.7) | 3 (9.1) / 2 (15.4) | 3 (21.4) / 2 (40) |  |
| **Surgery** |  |  |  |  |
| Tissue biopsy | 2 (4.7) / 5 (21.7) | 1 (3) / 1 (7.7) | 1 (7.1) / 1 (20) |  |
| Resection | 41 (95.3) / 18 (78.3) | 32 (97) / 12 (92.3) | 13 (92.9) / 4 (80) |  |
| **Total resection** |  |  |  |  |
| No | 7 (16.3) / 10 (43.5) | 5 (15.2) / 3 (23.1) | 3 (21.4) / 2 (40) |  |
| Yes | 36(83.7) / 13 (56.5) | 28 (84.8) / 10 (76.9) | 11 (78.6) / 3 (60) |  |
| **Recurrent resection** |  |  |  |  |
| No | 33(76.7) / 14 (60.9) | 25 (75.8) / 7 (53.8) | 11 (78.6) / 4 (80) |  |
| Yes | 10 (23.3) / 9 (39.1) | 8 (24.2) / 6 (46.2) | 3 (21.4) / 1 (20) |  |
|  | **Mean (SD)** | **Mean (SD)** | **Mean (SD)** |  |
|  | **Number (Percent)** | **Number (Percent)** | **Number (Percent)** |  |
| **Distress Thermometer** |  |  |  |  |
| Distress Mean (SD) | 5.4 (3.0) / 4.6 (2.8) | 4.3 (2.1) / 3.6 (2.3) | 5.0 (2.7) / 4.4 (3.9) | p = 0.209^F^ |
| DT ≥ 5 | 25 (58.1) / 16 (69.6) | 18 (41.9) / 7 (30.4) | 7 (16.3) / 1 (20) |  |
| **Family Problems** |  |  |  |  |
| Yes | 5 (11.6) / 2 (8.7) | 27 (18.2) / 2 (8.7) | 1 (7.1) / 2 (40) |  |
| No | 38 (88.4) / 21 (91.3) | 6 (81.8) / 21 (91.3) | 13 (92.9) / 3 (60) |  |
| **Practical Problems** |  |  |  |  |
| Yes | 5 (11.6) / 7 (30.4) | 10 (30.3) / 7 (30.4) | 2 (14.3) / 1 (20) |  |
| No | 38 (88.4) / 16 (69.6) | 23 (69.7) / 16 (91.3) | 12 (85.7) / 4 (80) |  |
| **Emotional Problems** |  |  |  |  |
| Yes | 34 (79.6) / 18 (78.3) | 21 (63.6) / 18 (78.3) | 6 (42.9) / 2 (40) |  |
| No | 9 (20.9) / 5 (21.7) | 12 (36.4) / 5 (21.7) | 8 (57.1) / 3 (60) |  |
| **Spiritual Problems** |  |  |  |  |
| Yes | 1 (2.3) / 2 (8.7) | 1 (3.0) / 2 (8.7) | 2 (14.3) / 0 (0) |  |
| No | 42 (97.7) / 21 (91.3) | 32 (97.0) / 21 (91.3) | 12 (85.7) / 5 (100) |  |
| **Physical Problems** |  |  |  |  |
| Yes | 35 (81.4) / 15 (65.2) | 27 (81.8) / 15 (65.2) | 11 (78.6) / 3 (60) |  |
| No | 8 (18.6) / 8 (34.8) | 6 (18.2) / 8 (34.8) | 3 (21.4) / 2 (40) |  |
| **GAD-2** |  |  |  |  |
| GAD-2 mean (SD) | 1.6 (1.5) / 1.6 (1.8) | 1.6 (1.9) / 0.2 (0.4) | 1.3 (1.8) / 2.0 (2.5) | p = 0.341^F^ |
| GAD-2 ≥ 3 | 9 (20.9) / 10 (43.5) | 6 (14.0) / 3 (13) | 2 (4.7) / 1 (20) |  |
| **PHQ-2** |  |  |  |  |
| PHQ-2 mean (SD) | 1.9 (2.0) / 1.2 (1.3) | 1.1 (1.3) / 1.2 (1.3) | 0.8 (1.1) / 2.2 (2.7) | p = 0.167^F^ |
| PHQ-2 ≥ 3 | 5 (11.6) / 9 (39.1) | 4 (9.3) / 1 (4.3) | 2 (4.7) / 2 (40) |  |
| **HHI** |  |  |  |  |
| HHI Mean (SD) | 46.3 (1.7) / 46.0 (1.7) | 45.0 (3.8) / 41.00 (5.1) | 44.5 (2.1) / 36.7 (9.1) | **p = 0.044^F^** |
| Missing Data N (%) | 0 / 0 | 3 (9.1) / 1 (7.7) | 9 (64.3) / 2 (40) |  |
| **Psychotherapeutic treatment** |  |  |  |  |
| Never | 32 (74.7) / 16 (69.6) | 25 (75.8) / 8 (61.5) | 10 (71.4) / 4 (80) |  |
| In the past | 9 (20.9) / 5 (21.7) | 4 (12.1) / 3 (23.1) | 2 (14.3) / 1 (20) |  |
| Currently in treatment | 2 (4.7) / 2 (8.7) | 4 (12.1) / 2 (15.4) | 2 (14.3) / 0 (0) |  |
| **Tranquilizers** |  |  |  |  |
| Never | 37 (86) / 18 (78.3) | 26 (78.8) / 12 (92.3) | 12 (85.7) / 5 (100) |  |
| Daily | 3 (7) / 5 (21.7) | 5 (15.2) / 1 (7.7) | 2 (14.3) / 0 (0) |  |
| Occasionally | 3 (7) / 0 (0) | 2 (6.1) / 0 (0) | 0 (0) / 0 (0) |  |
| **Coping Parameters** |  |  |  |  |
| 1 Expected treatment success | 2.5 (1.9) / 1.5 (0.5) | 4.1 (2.8) /  4.5 (0.9) | 4.2 (3.0) / 6.3 (3.2) | **p = 0.018^F^** |
| Missing Data N (%) | 0 / 0 | 5 (15.2) / 0 | 9 (64.3) / 2 (40) |  |
| 2 Expected side effects | 3.7 (1.9) / 2.7 (2.5) | 2.9 (0.7) / 4.5 (0.9) | 2.6 (0.9) / 3.0 (2.7) | p = 0.902^F^ |
| Missing Data N (%) | 0 / 0 | 5 (15.2) / 0 | 9 (64.3) / 2 (40) |  |
| 3 Subjective former resilience | 1.8 (0.5) / 1.0 (0.0) | 2.5 (1.0) / 1.8 (0.4) | 3.3 (2.6) / 2.0 (1.4) | p = 0.560^F^ |
| Missing Data N (%) | 9 (20.9) / 0 | 5 (15.2) / 0 | 9 (64.3) / 2 (40.0) |  |
| 4 Subjective current mental health status | 1.8 (0.5) / 4.5 (3.1) | 2.5 (1.0) / 4.2 (1.4) | 3.3 (2.6) / 4.3 (3.2) | p = 0.639^F^ |
| Missing Data N (%) | 0 / 0 | 5 (15.2) / 0 | 9 (64.3) / 2 (40) |  |
| 5 Subjective ability to handle the disease | 2.4 (1.9) / 2.5 (0.9) | 2.4 (1.1) / 3.5 (1.3) | 2.4 (0.9) / 6.0 (3.5) | p = 0.191^F^ |
| Missing Data N (%) | 0 / 0 | 5 (15.2) / 0 | 9 (64.3) / 2 (40) |  |
| 6 Experienced social support | 1.3 (1.6) / 1.2 (0.3) | 1.6 (0.7) / 2.2 (0.3) | 1.4 (0.6) / 1.7 (1.2) | p = 0.156^F^ |
| Missing Data N (%) | 0 / 0 | 5 (15.2) / 0 | 9 (64.3) / 2 (40) |  |

Abbreviations: T, measurement time point; SD, Standard deviation; F, Fisher’s exact test, DT, Distress Thermometer, GAD-2, Generalized Anxiety Disordern2-item; PHQ-2, Patient Health Questionnaire-2; HHI, Herth Hope Index
